# Network Analysis for Uncovering the Relationship between Host Response and Clinical Factors to Virus Pathogen: Lessons from SARS-CoV-2

**DOI:** 10.3390/v14112422

**Published:** 2022-10-31

**Authors:** Milan Sova, Milos Kudelka, Milan Raska, Jan Mizera, Zuzana Mikulkova, Marketa Trajerova, Eliska Ochodkova, Samuel Genzor, Petr Jakubec, Alena Borikova, Ladislav Stepanek, Petr Kosztyu, Eva Kriegova

**Affiliations:** 1 Department of Pulmonary Diseases and Tuberculosis, Faculty of Medicine and Dentistry, Palacký University and University Hospital Olomouc, 779 00 Olomouc, Czech Republic; 2 Department of Respiratory Medicine, University Hospital, 625 00 Brno, Czech Republic; 3 Department of Computer Science, Faculty of Electrical Engineering and Computer Science, VSB-Technical University of Ostrava, 701 03 Ostrava, Czech Republic; 4 Department of Immunology, Faculty of Medicine and Dentistry, Palacký University and University Hospital Olomouc, 779 00 Olomouc, Czech Republic; 5 Department of Occupational Medicine, University Hospital Olomouc, 779 00 Olomouc, Czech Republic

**Keywords:** patient similarity network, multivariate data analysis, COVID-19 severity, minimal immune signature, data visualisation, IgM and IgG levels

## Abstract

Analysing complex datasets while maintaining the interpretability and explainability of outcomes for clinicians and patients is challenging, not only in viral infections. These datasets often include a variety of heterogeneous clinical, demographic, laboratory, and personal data, and it is not a single factor but a combination of multiple factors that contribute to patient characterisation and host response. Therefore, multivariate approaches are needed to analyse these complex patient datasets, which are impossible to analyse with univariate comparisons (e.g., one immune cell subset versus one clinical factor). Using a SARS-CoV-2 infection as an example, we employed a patient similarity network (PSN) approach to assess the relationship between host immune factors and the clinical course of infection and performed visualisation and data interpretation. A PSN analysis of ~85 immunological (cellular and humoral) and ~70 clinical factors in 250 recruited patients with coronavirus disease (COVID-19) who were sampled four to eight weeks after a PCR-confirmed SARS-CoV-2 infection identified a minimal immune signature, as well as clinical and laboratory factors strongly associated with disease severity. Our study demonstrates the benefits of implementing multivariate network approaches to identify relevant factors and visualise their relationships in a SARS-CoV-2 infection, but the model is generally applicable to any complex dataset.

## 1. Introduction

The analysis of complex datasets is a major challenge in all branches of medicine, as these datasets often include diverse clinical, demographic, laboratory, and personal data. In addition, there is considerable heterogeneity between patients in terms of clinical manifestations, the presence or absence of multiple individual factors contributing to clinical symptoms, and longitudinal changes in multiple factors throughout the disease course. Univariate analysis (e.g., one laboratory factor versus one clinical factor) is still commonly used in this context, as shown, for example, by studies analysing the relationship between the host immune response and clinical factors in a severe acute respiratory syndrome 2 (SARS-CoV-2) infection [1,2,3,4,5]. However, univariate analysis cannot reveal information about the complex relationships among multiple factors. Moreover, it violates the independence assumption for correlated factors.

Therefore, multivariate analyses are a preferable approach when analysing complex datasets, providing a more realistic basis for robust and accurate clinical decisions [6]. Moreover, multivariate analysis enables the assessment of the contribution of multiple factors concerning one or more clinical factors to reflect reality, reveal relationships between the factors analysed, and reduce the bias of univariate patient characteristics across studies [6,7,8,9]. Nowadays, several multivariate approaches are available that consider complex, multidimensional relationships between factors. These approaches can be divided into four groups according to the objectives of the analysis [10]: (i) comparison of treatment groups influenced by experimental treatment structure using multivariate analysis of variance (MANOVA); (ii) dimensionality reduction techniques such as principal component analysis (PCA); (iii) discriminate techniques such as canonical discriminant analysis (CDA); and (iv) cluster analysis—there are many different algorithms that produce different-sized clusters.

However, these traditional multivariate approaches have several limitations in analysing complex datasets [11], particularly in terms of interpreting the data and visualising the contribution of individual factors. The interpretation of the results of multivariate analysis is generally a difficult task. Examples include the interpretation of derived factors and the number of components obtained by PCA or the interpretation of clusters and their number and size produced by one of the many cluster analysis techniques. There are also limitations in the visualisation options, as complex datasets usually work with many factors (dozens or more are common). For visualisation, however, two or three factors are needed at most. The multidimensional data space must, therefore, be transformed into 2D or 3D so that the result is understandable to the observer. This problem is then solved by dimension reduction, either by feature extraction, where PCA is an example, or by feature selection, i.e., choosing two or three factors. This is followed by visualisation in 2D (e.g., scatter plots) or 3D. Although the result is usually clear, it can be somewhat confusing. In both cases, the observer loses some information, the lack of which is most evident when studying the details of individual points in the data space, in our case, patient profiles.

One innovative multivariate approach to analyse complex biomedical datasets is a network approach, which is based on the realisation that the similarities of the patient profiles are essential for a reasonable interpretation for us as observers [12,13,14,15,16]. This relationship is pairwise, and traditional visualisations lack an exact representation of it. In traditional visualisation, we perceive this relationship as a metric distance of points in the visual form. However, when reducing the dimension, this distance may not represent what describes reality and what we deduce from the visualisation [12,17,18]. As explained later, in this approach, networks in which a sufficiently high similarity of a pair of patients (more precisely, their profiles) is expressed straightforwardly by their ties in the visualisation, and the strength of this tie represent the degree of this similarity. In this respect, networks are a tool that allows visualisation of what is missing in traditional approaches. The main and major advantage of multivariate network analysis is its depth of insight due to the visualisation, allowing us to interpret the data and extract meaning from it, regardless of the type of data.

Here, we investigated the applicability of network analysis for uncovering the relationship between the host immune response and clinical and laboratory factors of virus infections using a SARS-CoV-2 infection as an example. Despite the growing number of studies on coronavirus disease (COVID-19), interpreting the data and drawing meaningful conclusions from the data are challenging. We evaluated a comprehensive dataset from patients infected with the SARS-CoV-2 virus during the first and second COVID-19 waves (period from March to November 2020) of the pandemic in the Czech Republic, enabling us to compare obtained data with published studies. The main objective of this study was to compare the results obtained from univariate analysis and multivariate network analysis, which provides visualisation as an analytical approach to help interpret complex data and identify minimal immune signals useful as a potential predictor of disease severity or persistence of complications.

## 2. Materials and Methods

### 2.1. Patients

The study cohort consisted of 250 patients (124 men and 126 women; mean age ±SD: 53.5 ± 14.0 years) infected with the SARS-CoV-2 virus between March and November 2020. The predominant SARS-CoV-2 variants detected during this period, corresponding to the first and second waves of COVID-19 in the Czech Republic, were B.1, B.1.1.266 and B.1.258 [18]. None of the patients enrolled in this study were vaccinated at the time of the SARS-CoV-2 infection and sampling. Of the patients, 83 (33%) had been admitted to the hospital; 80 (32%) had anosmia/ageusia, and 111 (44%) had pneumonia. Clinical evaluation, lung function data, and samplings (peripheral blood) were performed four to eight weeks after a positive SARS-CoV-2 diagnostic PCR test. Fourteen patients were excluded from analyses because of missing specimens for flow cytometry. For more details on clinical characteristics, see Table 1.

### 2.2. Characteristics of Analysed Data

For enrolled COVID-19 patients, ~85 immunological (cellular and humoral) and ~70 clinical factors available at the date of sampling were analysed.

All patients underwent a chest X-ray and pulmonary function tests, including vital capacity, forced vital capacity, forced expiratory volume in 1 s, forced expiratory flow/vital capacity ratio, peak exploratory flow, total lung capacity, carbon monoxide diffusing capacity (DLCO), and carbon monoxide transfer coefficient. In the case of residual findings on chest X-rays indicating the persistence of lung interstitial changes, high-resolution chest computed tomography was subsequently performed. In addition, clinically relevant medical history data related to COVID-19, e.g., history of hospitalisation, pneumonia, persistent dyspnoea, symptoms, and anosmia/ageusia (partial/complete), were collected in all patients.

Among immunological factors, the main immune cell populations and subpopulations and their activation in peripheral blood were determined using flow cytometry. Whole blood samples were prepared for eight-colour flow cytometry as previously described [19]. Isotype-matched conjugated irrelevant antibodies and fluorescence minus one controls were used. All antibodies and isotype controls used were purchased from BioLegend (San Diego, CA, USA). For flow cytometry analysis, BD FACSCanto II (BD Biosciences, San José, CA, USA) was used. For data evaluation, FlowJo v10.8.1 (BD Biosciences, Franklin Lakes, NJ, USA) was used. The main blood cell populations were identified using the sequential gating strategy after exclusion of doublets (FSC-A/FSC-H) as follows: main immune cell populations (lymphocytes, LYM; monocytes, MON; neutrophils, NEU) were identified by FSC-A/SSC-A, T-lymphocytes (T-LYM; CD3+ LYM), NK cells (CD3− /CD16+ CD56+ LYM); B-LYM (CD19+ /CD3− LYM), immature B-cells (CD19+ /CD27− /CD38+), eosinophils (EOS; CD49d+ /CD16− /CD15+), and basophils (BAS; CD203c+ /CD123+ /FcεRIα+). A minimum of 10,000 events were collected. A cut-off of 500 events was used to evaluate activation markers and immune checkpoint molecules (CD69, HLA-DR, PD-1, and CTLA-4). The data are presented either as percentages of immune cell singlets (LYM, MON, NEU, EOS, and BAS) or percentages of parental populations (T-LYM, B-LYM, and NK cells from LYM; immature B-LYM from B-LYM).

The serum levels of IgG and IgM were assessed by ELISA using the recombinant SARS-CoV-2 RBD Wuhan variant. In addition, 96-well plates (Nunc) were coated with RBD (50 ng/well) overnight at 4 °C, washed, and blocked with 1% BSA/PBS/Tween-20 for three hours at room temperature (RT). Sera were diluted 1:1000 in blocking buffer (in triplicates), incubated overnight at 4 °C, washed, and incubated with secondary rabbit anti-human IgG and IgM antibodies conjugated with horseradish peroxidase (Sigma-Aldrich, Saint-Louis, MO, USA) diluted 1:10,000 in blocking buffer for three hours at RT. The signal developed with OPD-H_2_O_2_ was measured at 492 nm and expressed as optical density. The threshold of positivity was based on a comparison with a cohort of noninfected subjects sampled before the SARS-CoV-2 pandemic.

### 2.3. Patient Similarity Network (PSN)

To visually study and assess the relationships between patient profiles and groups of similar patients, we need to convert the patient data into a patient similarity network (PSN). This conversion must work with multiple aspects. The first is the selection of small combinations of such (immunological and clinical) factors that are relevant for similarity assessment. For example, if we have measured 150 factors, we can expect that most of them are noise for the problem we intend to solve. The second aspect is the method of measuring similarity, which must be chosen based on domain knowledge; we use a so-called Gaussian function that converts a distance metric to a similarity metric from an interval [0, 1], as in approaches similar to ours; before applying the Gaussian function, the values of each factor were re-scaled to the same interval. The third aspect is the application of a method that constructs a network from the selected factor in the patient profile; here, we use the LRNet method [17, LRNet application: https://homel.vsb.cz/~kud007/lrnet_files (accessed on 30 October 2022)], which both preserves the essence of the relationships in the original data and reveals not-so-obvious features hidden in the data. The last two aspects are the assessment of the quality of the network in terms of its density (degree of connectivity) and the separation of the parts into clusters of different sizes; here, we use a method to detect clusters in the network (Louvain modularity [20]). In particular, we measure the quality of the separation between clusters using two characteristics, which are the Louvain modularity from interval [−0.5, 1] and silhouette from interval [−1, 1]. Simply put, the higher the modularity, the better clusters of similar patients are separated in the network. The higher the silhouette (positive values), the more reliably individual patients belong to the cluster to which they have been automatically assigned.

Other descriptive characteristics are based on the measurement of univariate statistics within clusters. Here, we assume that patients should have similar values in each factor in a particular cluster, and at least for some factors, their values should differ between clusters. Arithmetic means, standard deviations, and confidence intervals are computed for all factors in the clusters to assess the satisfaction of both assumptions.

Using computers makes it possible to work with all of the above aspects at the same time by having a computer program automatically generating networks for small but varying combinations of factors. The quality of the generated networks is automatically measured based on the abovementioned characteristics. As a result, the combination of factors and networks proposed by the computer program are of the highest quality concerning all mentioned aspects. From these proposals, a network is then manually selected that captures the domain knowledge and, thus, also meets the requirements for clinical applicability. In our case, we considered and investigated combinations of 3–5 factors out of 85 immunological factors and the best combination based on the network quality measures and clinical relevance was nominated. The modularity of the nominated network was 0.616, and 82.6% of patients had a positive silhouette expressing their unambiguous placement in one of the clusters. In addition, a selection from 70 clinical factors was used for visualisation within the network.

### 2.4. Statistics

The Kruskal–Wallis one-way ANOVA test in three or more groups and the Wilcoxon–Mann–Whitney test between the two groups were used to compare the distribution of immune cells, their activation, IgG/IgM levels, and clinical factors. The achieved levels of statistical significance (*p*-values) were carried out using the R language (3.6.1) in the R-Studio 1.2.× programming environment (http://www.r-project.org/ accessed on 1 October 2022). *p*-values of <0.05 were considered significant. The data are presented as a mean and 95% confidence interval (CI).

## 3. Results

### 3.1. Univariate Analysis of Obtained Data

**Figure 1:**
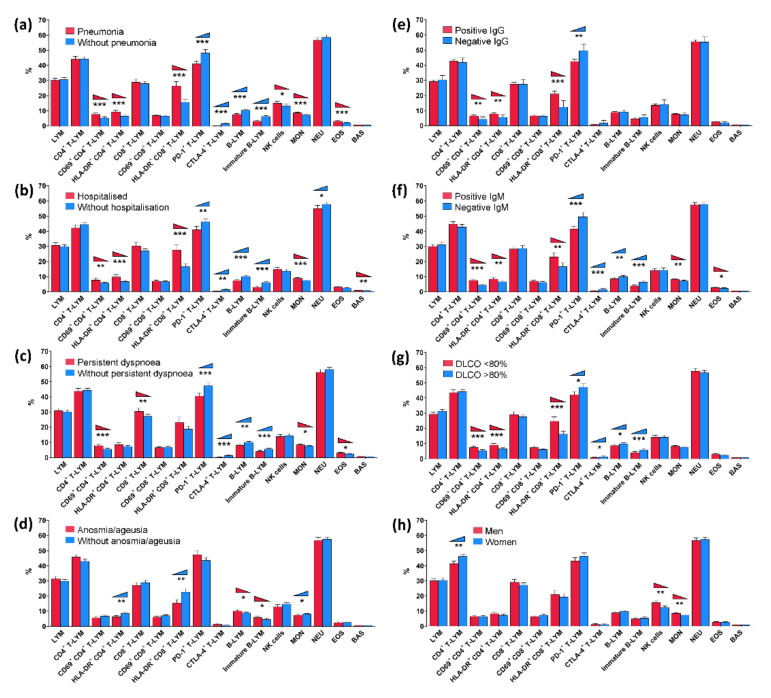
Profile of circulating immune cells four to eight weeks post-COVID-19 in patients subdivided according to: (**a**) pneumonia, (**b**) hospitalisation history, (**c**) persistent dyspnoea at the post-COVID-19 check-up, (**d**) anosmia/ageusia, (**e**) positive serum IgG levels at post-COVID sampling, (**f**) positive serum IgM levels at post-COVID sampling, (**g**) DLCO (%predicted) at post-COVID sampling, and (**h**) gender. Factors (**c**) to (**g**) refer to a period of four to eight weeks post-COVID-19. LYM: lymphocytes; MON: monocytes; NEU: neutrophils; EOS: eosinophils; BAS: basophils; DLCO: carbon monoxide diffusing capacity. Bars show the mean value, and whiskers the 95% confidence interval. * *p* < 0.05; ** *p* < 0.01; *** *p* < 0.001.

We first examined changes in circulating immune cells and other factors associated with SARS-CoV-2 infections in patients with mild and severe disease manifestations, defined by the presence of pneumonia (Figure 1a) and hospitalisation history (Figure 1b), using univariate analysis.

In addition, we assessed the distribution of circulating immune cells in patients subgrouped according to persistent dyspnoea at the post-COVID-19 check-up (Figure 1c), anosmia/ageusia (Figure 1d), serum IgG levels at post-COVID sampling (Figure 1e), serum IgM levels at post-COVID sampling (Figure 1e), DLCO (%predicted) at post-COVID sampling (Figure 1g), and gender (Figure 1h). The data are also presented in the table format with a corresponding *p*-value and 95%CI (Table 2).

For each condition/clinical factor of interest from the comprehensive dataset, statistical significance is calculated and reported as the difference in the value of a given factor (higher, lower, or no difference) between the subgroups of patients studied. For example, our patients with COVID-19 who had confirmed pneumonia had elevated percentages of activated CD8+ T-lymphocytes, activated CD4+ T-lymphocytes, NK cells, monocytes, and eosinophils but lower percentages of B-lymphocytes and immature B-lymphocytes as well as lower percentages of T-lymphocytes expressing checkpoint molecules PD-1 and CTLA-4 at post-COVID sampling compared to patients without pneumonia. No difference in CD8+ T-lymphocytes, CD4+ T-lymphocytes, neutrophils, and basophils was detected between these two studied groups (Table 2c, Figure 1c). Regarding another example of COVID-19 patients with anosmia/ageusia, elevated percentages of B-lymphocytes and immature B-lymphocytes but lower percentages of activated CD8+ T-lymphocytes, activated CD4+ T-lymphocytes, and monocytes were detected when compared to patients without anosmia/ageusia. No difference in the distribution of lymphocytes, CTLA-4+ T-lymphocytes, neutrophils, eosinophils, and basophils was detected between patients with and without anosmia/ageusia. In addition, a trend to higher percentages of CD4+ T-lymphocytes, PD-1+ T-lymphocytes, and lower NK cells was detected in patients reporting anosmia/ageusia; however, the difference did not reach significance. All other conditions and factors could be evaluated in the same manner.

### 3.2. Multivariate Patient Similarity Network Analysis (PSN)

**Figure 2:**
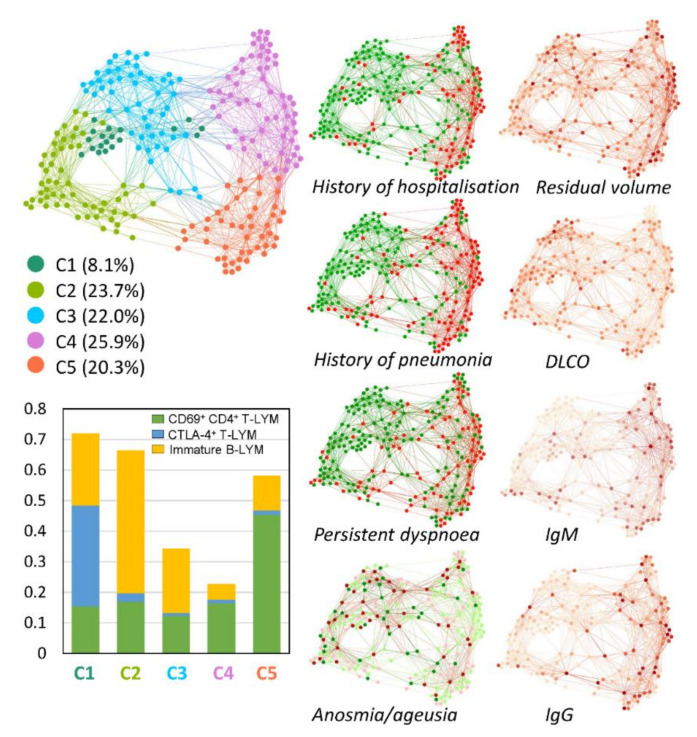
PSN analysis. The PSN identified five patient clusters (C1–C5) associated with COVID-19 severity based on a minimal immune signature (CD69+ CD4+ T-lymphocytes, CTLA-4+ CD4+ T-lymphocytes, and immature B-LYM; for their distribution within clusters, see the bar chart). Cluster 1 (C1, dark green), cluster 2 (C2, green), and cluster 3 (C3, blue) were predominantly associated with mild disease; cluster 4 (C4, violet) and cluster 5 (C5, orange) with severe disease. Dark red/green network vertices represent individual patients with/without a history of pneumonia, hospitalisation, persistent dyspnoea, and anosmia/ageusia (red/green = women/men; light red/green were patients not suffering from anosmia/ageusia; dark red/green were patients suffering from anosmia/ageusia). Pulmonary function and serum IgM and IgG levels in individual patients four to eight weeks post-COVID-19 are indicated by the intensity of red (light for the lowest values and dark for the highest values). C: cluster; LYM: lymphocytes; DLCO: diffusing capacity from carbon monoxide in the lungs.

In the next step, we applied a multivariate unsupervised PSNs approach utilising the clustering based on the similarity in the distribution of circulating immune cells, their immunophenotypes, and clinical, functional, and laboratory factors within patients, creating a network and subsequent visualisation of other factors of interest (Figure 2). In it, we can see the top left panel containing colour-coded clusters of patients that are similar to each other (connected by a tie) in three factors that proved crucial when converting the dataset into the network. As additional information, the averages of the patient profiles in each cluster are shown here in the bottom left panel as a bar chart. Due to the different colouring of the network on the right panel, we have the same network in several variants providing different information. What is particularly evident in the visualisations is that the data are heterogeneous, and the trends captured in the data are more important than the statistical values.

Despite numerous deregulated immune populations and their activation markers across the cohort (Figure 1), clustering based on a percentage of activated (CD69+) CD4+ T-lymphocytes, CTLA-4+ T-lymphocytes, and immature B-lymphocytes (CD19+ CD27− CD38+(bright)) revealed the best subdivision of patients based on COVID-19 severity and lung function status four to eight weeks after initial infection (Figure 2).

As visualised in Figure 2, a majority of patients with a high percentage of activated CD4+ T-lymphocytes and low percentages of CTLA-4+ T-lymphocytes and immature B-lymphocytes had pneumonia, a history of hospitalisation, impaired lung function, and persistent dyspnoea at the post-COVID-19 check-up. In addition, PSNs allow multiple factors to be displayed simultaneously in the network, as demonstrated, for example, in a subanalysis of anosmia/ageusia in women and men who had moderate and severe manifestations of COVID-19 (Figure 2, lower part).

## 4. Discussion

The healthcare system generates a significant amount of data. In addition, a large amount of omics data has been collected in the literature and public databases. However, we often need to better understand these data, extract the meaning from them, and make the most rational use of their potential to generate the knowledge we need. This is also important for uncovering relationships between many laboratory, clinical, and personal factors in complex diseases/conditions, which can help in data interpretation and better management of patients. The situation is also similar for the host response to individual pathogens, where not a single factor but a combination of multiple factors contributes.

This study focused on uncovering the host response to the SARS-CoV-2 virus as an example of a complex condition. We studied the relationship between a hundred clinical, demographic, laboratory, and personal factors available from 250 patients with COVID-19 by applying both univariate analyses and multivariate network analyses. All patients were sampled during the first and second COVID-19 waves of the SARS-CoV-2 pandemic. At that time, patients were monitored thoroughly, with very good communication between physicians and patients, and none of the patients had been vaccinated. The advantage of the patient cohort used is also the significant amount of published data from different populations that are currently available for comparison.

The majority of published studies on patients with COVID-19 still rely on univariate analysis [1,2,3,4,5]. This type of analysis enables us to understand the distribution of values for a single condition, but it cannot understand the relationship between two and more variables. As shown by our study, the visualisation of univariate analysis is easy for the observer to understand; they can see the average ranking of the laboratory factors for each pair of clinical factors/conditions being compared, as well as which patient subgroup has the higher average value. Additional information here is the CIs of the significance of the differences. The first problem is that if we want to study a single patient and its similarity to another, we will not find a solution in this visualisation. The second issue is the overall view of the patient dataset, which raises many questions. For example, how can we see whether one or more factors cluster patients in one place? How large is a subset of factors sufficient to translate the patient dataset into an understandable visual form so that we can look for answers to multiple similar questions? Although the profiles of immune cells vary between comparisons, it is impossible to identify the most significant combination of factors (here, immune cell subpopulations) associated with disease manifestations. As shown in our dataset, the univariate analysis revealed factors associated with a particular condition, such as pneumonia, anosmia/ageusia, or hospitalisation, but, for example, such analysis does not answer questions about whether patients with pneumonia also had anosmia/ageusia or were hospitalised, and which combination of factors are associated with particular patient subgroups. Generally, the results of univariate analysis lack deeper insight into the relationships between the spectrum of factors and their relevance to reality, as well as they are not being suitable for patient groups that are very heterogeneous in terms of phenotypes, immune responses, and clinical manifestations.

Therefore, we introduced in this study an innovative multivariate network analysis to assess the relationship between host response and SARS-CoV-2 infection. Over the years, there has been increasing evidence of the benefits of multivariate analysis in complex diseases [14], as the outcome may be caused by multiple factors, and there may be several different disease phenotypes with different factors/mechanisms involved. We applied a network analysis called PSN that constructs a network from vector data and then clusters the patients based on the similarity in their factors [12,21]. It is suitable for any data (clinical, laboratory, demographic, and functional) of any type (binary and continuous) and cohort size. This makes it highly suitable for the analysis of biomedical datasets [12,13,14,15,16,22]. The advantage of using a PSN network and its visualisation also helps to study and identify the relevant factors for the given conditions, which allow for the subgrouping of patients. Indeed, this multivariate analysis of our real-world cohort identified a minimal immune signature consisting of a high percentage of activated CD4+ T-lymphocytes and low percentages of CTLA-4+ T-lymphocytes and immature B-lymphocytes that were strongly associated with disease severity and manifested even four to eight weeks after COVID-19. Other immune features showed high variability across the cohort, indicating significant heterogeneity in the immune response in individual patients.

Our data are in line with other studies in which a higher representation of activated (CD69+) interferon-γ-producing CD4+ T-lymphocytes was detected in hospitalised COVID-19 patients with pneumonia [23]. Dysregulation in circulating B-cell subpopulations, particularly a reduced number of immature B-cells and normalisation within three to six months of convalescence, has been recently reported in patients with severe COVID-19 [24,25]. Our data indicates that the B-cell disturbance observed in acute severe COVID-19 is extended for at least four to eight weeks after acute infection. This appears to be a response similar to the forced maturation of B-cells towards plasmablasts, achieving up to 30% of the total peripheral blood B-cells, reported in a subgroup of COVID-19 patients [26]. A patient subset (8%) with the mild disease had high levels of checkpoint inhibitor CTLA-4 on T-cells, probably involved in the downregulation of immune activation during COVID-19-related cytokine storms. In the presented study, the patients with severe disease had lower CTLA-4 expression on T-cells, allowing us to hypothesise that in such patients, less controlled activation of T-cells could support the development of severe inflammation in the lungs. In contrast, other authors hypothesised the inhibition of CTLA-4 as the potential therapeutic approach supporting antiviral T-cell activity and inhibiting T-cell exhaustion [27].

Furthermore, the PSN multivariate analysis indicated that anosmia/ageusia was more frequent with mild disease, consistent with previous studies [28]. Moreover, in our cohort, more women than men suffered from olfactory and taste dysfunction. This may be because women are more sensitive to altered olfactory perception than men, as evidenced in a recent meta-analysis [29]. Nevertheless, our knowledge of sex-related differences in the expression of ACE2 and TMPRSS2, two key receptors required for SARS-CoV-2 entry, on non-neural-type sustentacular cells in the olfactory epithelium, which are responsible for SARS-CoV-2-related taste and smell impairments, is very limited and is largely based on animal models [30].

In addition, visualisation of clinical and laboratory factors in patient clusters detected by PSN showed that a history of severe COVID-19 was associated with higher levels of IgM and IgG compared with mild disease. There is evidence that both IgM and IgG can be detected around the same time (~2 weeks) after a SARS-CoV-2 infection, with the development of the class-switched, high-affinity IgG response for long-term immunity and immunological memory [31]. Recent findings have demonstrated a significant correlation of memory B-cells with both IgG1 and IgM responses to the SARS-CoV-2 spike protein receptor-binding domain in most seropositive subjects [32]. IgM+ memory cells are detectable over three months together with switched memory cells with somatic hypermutations, which increase in frequency for several months after the onset of symptoms and persist stably for at least six to eight months [33]. This may explain the high levels of IgM and IgG in our patients with severe disease two months post-COVID-19. Our observation of low levels of immature B-cells in severe disease agrees with the report that patients with IgM memory B-cell depletion died [32] and further highlights the statement that B-cells, particularly memory IgM B-cells, are a critical indicator of disease severity and resolution.

Our study showed several advantages of PSN in a clinically well-characterised real-world cohort of Caucasian patients with the full spectrum of clinical variability of a SARS-CoV-2 infection. Moreover, our study shows that the PSN approach allows for the visualisation and interpretation of relevant information in complex datasets while maintaining the interpretability and explainability of outcomes for clinicians and patients, which is impossible in binary comparisons. In comparison to traditional multivariate approaches, the presented PSN has a big advantage of independence on a pre-set number and size of the clusters (=patients with similar profiles); it allows the integration of diverse data types and handling of sparse data, selection of the most relevant combinations of factors, the possibility to reanalyse each patient cluster, and excellent model interpretability [12,22,34]. Moreover, this approach allows the comparison of datasets obtained for other pathogen strains/time points/conditions. Finally, the obtained models may be improved by adding novel patients and/or factors that may lead to a more precise model. However, we do not consider using networks as the only option and rather see it as an alternative or important complement to traditional visualisation methods.

This study also has limitations. First, we analysed only a group of patients from the first and second waves of the COVID-19 pandemic who were not vaccinated. Currently, immune profiles and other analysed factors may be affected by the vaccination, which has been performed in approximately two-thirds of the population [35,36], reinfections with SARS-CoV-2, and the emergence of other virus strains [37]. Second, our study focused mainly on patients with severe disease manifestations, which does not allow us to compare with patients infected with the current Omicron variant, because most patients have mild to moderate disease manifestations that do not require hospitalisation. Hospitalised patients infected with the Omicron variant are mainly those with comorbidities or who are at risk or those who refuse vaccination and participation in clinical and research studies [35,38,39]. However, the used cohort of patients enabled us to compare the obtained data with previously published studies from the same period.

## 5. Conclusions

By exploring and visualising multiple variables from our SARS-CoV-2 real-world dataset using an unsupervised network analysis approach PSN, we have shown that it is possible to obtain (i) a detailed model of the relationships among multiple factors and (ii) actionable and interpretable observations in real-world datasets. Specifically, we identified a minimal immune signature consisting of three parameters: a high percentage of activated CD4+ T-lymphocytes, a low percentage of CTLA-4+ T-lymphocytes, and a low percentage of immature B-lymphocytes, that were strongly associated with COVID-19 severity expressed as the need for hospitalisation, pneumonia, impaired lung functions, and a persistent dyspnoea four to eight weeks after the COVID-19 diagnostic PCR test. In addition, visualisation of clinical and laboratory factors in patient clusters detected by PSN showed that the minimal immune signature associated with a history of severe COVID-19 disease also associated with higher levels of IgG and IgM and impaired lung function four to eight weeks after COVID-19. Furthermore, anosmia/ageusia in the first and second waves of COVID-19 was more frequently associated with a mild course of disease, and more women than men had olfactory and gustatory dysfunction. Based on results consistent with published findings from the first and second waves of COVID-19, we have shown that it is possible to find a model that can be translated into a visual and easily interpretable form.

Taken together, this study demonstrates the advantages of using multivariate analysis over univariate analysis when studying complex datasets. Although we have presented the advantages of network analysis to study the host response to a SARS-CoV-2 infection, the model is generally applicable to any complex dataset. Therefore, network analysis may be important for uncovering the relationship between many laboratory, clinical, and personal factors in complex diseases/conditions, as extracting meaning from complex datasets can help with data interpretation and better patient management.

## Figures and Tables

**Table 1:** Basic characteristics of enrolled patients with COVID-19.

Factors	No of the Patients (%)	
Gender (men/women)	124/126	
Age, median years (min-max)	55 (19–87)	
Serum IgG, median (min-max) (AU/mL)	118 (6.21–390)	
Serum IgM, median (min-max) (AU/mL)	5.65 (0.06–111)	
**Comorbidities**		
Pulmonary arterial embolisation	10 (4%)	
Diabetes mellitus	31 (12%)	
Ischemic heart disease	12 (5%)	
**Medical history related to COVID-19**		
Hospitalisation	82 (33%)	
Pneumonia	111 (44%)	
Anosmia/ageusia	80 (32%)	
Pulmonary interstitial changes	29 (12%)	
Systemic glucocorticoid therapy	23 (9%)	
Persistent dyspnoea	89 (36%)	
Persistent cough	61 (24%)	
**Pulmonary Function Tests**	**Measured** **±** **SD**	**Percentage of** **Predicted Values** **±** **SD**
VC (l)	3.91 ± 1.10	101.3 ± 16.81
FVC (l)	3.88 ± 1.10	104.19 ± 17.28
FEV1 (l)	3.12 ± 0.89	101.89 ± 17.15
FEV1/VC	-	80.06 ± 6.37
PEF (l/min)	7.30 ± 1.99	96.88 ± 19.33
TLC (l)	6.33 ± 1.34	104.77 ± 15.95
DLCO (l/s)	7.43 ± 2.33	80.55 ± 17.21
KCO (l/s)	1.33 ± 0.25	88.19 ± 15.37

VC—vital capacity, FVC—forced vital capacity, FEV1—forced expiratory volume in 1 s, FEV1/VC forced expiratory flow/vital capacity ratio, PEF—peak exploratory flow, TLC—total lung capacity, DLCO—carbon monoxide diffusing capacity, KCO—carbon monoxide transfer coefficient, SD—standard deviations.

**Table 2:** Distribution of circulating immune cells four to eight weeks post-COVID-19 in patients subdivided according to (**a**) pneumonia, (**b**) hospitalisation history, (**c**) persistent dyspnoea at the post-COVID-19 check-up, (**d**) anosmia/ageusia, (**e**) positive serum IgG levels at post-COVID sampling, (**f**) positive serum IgM levels at post-COVID sampling, (**g**) DLCO (%predicted) at post-COVID sampling, and (**h**) gender. Factors (**c**) to (**g**) refer to a period of four to eight weeks post-COVID-19. Significant *p*-values are marked in bold.

	(a) Pneumonia			(b) Hospitalisation		
Distribution of Immune Cells [%]	No	Yes	*p*-Value	No	Yes	*p*-Value
	Mean (95% CI)	Mean (95% CI)		Mean (95% CI)	Mean (95% CI)	
Lymphocytes (LYM)	30.8 (29.4–32.3)	30.3 (28.9–31.8)	0.856	30.4 (29.2–31.6)	31.1 (29.3–33.0)	0.534
CD4+ T-LYM	44.4 (43.0–45.9)	44.2 (42.3–46.1)	0.746	45.1 (43.7–46.4)	42.8 (40.6–44.9)	0.127
CD69+ CD4+ T-LYM	5.38 (4.66–6.11)	7.77 (6.85–8.70)	**<0.001**	5.77 (5.12–6.42)	7.75 (6.57–8.93)	**0.004**
HLA-DR+ CD4+ T-LYM	6.58 (5.87–7.29)	9.39 (8.27–10.5)	**<0.001**	6.78 (6.15–7.41)	9.89 (8.49–11.3)	**<0.001**
CD8+ T-LYM	28.2 (26.6–29.7)	29.1 (27.1–31.1)	0.638	27.5 (26.2–28.9)	30.8 (28.3–33.3)	0.051
CD69+ CD8+ T-LYM	6.63 (6.00–7.26)	7.00 (6.44–7.57)	0.140	6.68 (6.13–7.23)	7.01 (6.34–7.69)	0.221
HLA-DR+ CD8+ T-LYM	15.8 (13.9–17.7)	26.6 (23.5–29.6)	**<0.001**	16.8 (15.0–18.6)	28.0 (24.4–31.7)	**<0.001**
PD-1+ T-LYM	48.3 (46.0–50.6)	41.1 (39.2–43.0)	**<0.001**	46.9 (44.8–49.0)	41.6 (39.4–43.8)	**0.008**
CTLA-4+ T-LYM	1.56 (0.95–2.17)	0.29 (0.20–0.39)	**0.001**	1.35 (0.83–1.87)	0.33 (0.19–0.46)	**0.007**
B-LYM	10.5 (9.77– 11.2)	7.72 (7.05–8.40)	**<0.001**	10.2 (9.51–10.8)	7.50 (6.71–8.28)	**<0.001**
Immature B-LYM	6.39 (5.69–7.09)	3.20 (2.45–3.94)	**<0.001**	6.03 (5.38–6.68)	2.91 (2.06–3.76)	**<0.001**
NK cells	13.4 (12.3–14.5)	15.2 (13.9–16.5)	**0.036**	13.8 (12.8–14.8)	15.0 (13.4–16.5)	**0.221**
Monocytes (MON)	7.34 (6.96–7.73)	8.80 (8.30–9.30)	**<0.001**	7.49 (7.14–7.84)	8.97 (8.37–9.58)	**<0.001**
Neutrophils (NEU)	58.6 (57.0–60.2)	56.8 (55.1–58.5)	0.148	58.8 (57.4–60.2)	55.9 (53.7–58.1)	0.042
Eosinophils (EOS)	2.19 (1.91–2.47)	3.21 (2.67–3.75)	**0.001**	2.42 (2.13–2.71)	3.07 (2.43–3.72)	**0.082**
Basophils (BAS)	0.69 (0.64–0.74)	0.75 (0.68–0.82)	0.124	0.68 (0.63–0.73)	0.79 (0.71–0.86)	**0.005**
	**(c) Persistent Dyspnoea**			**(d) Anosmia/Ageusia**		
**Distribution of Immune Cells**	**No**	**Yes**	***p*** **-Value**	**No**	**Yes**	***p*** **-Value**
**[%]**	**Mean (95% CI)**	**Mean (95% CI)**		**Mean (95% CI)**	**Mean (95% CI)**	
Lymphocytes (LYM)	30.4 (29.0–31.7)	31.1 (29.5–32.7)	0.589	30.1 (28.9–31.4)	31.7 (30.0–33.4)	0.295
CD4+ T-LYM	44.5 (43.1–46.0)	44.0 (42.0–45.9)	0.908	43.4 (41.9–44.9)	46.1 (44.5–47.8)	0.077
CD69+ CD4+ T-LYM	5.57 (4.84–6.31)	7.88 (6.96–8.81)	**<0.001**	6.78 (6.03–7.52)	5.65 (4.70–6.60)	0.093
HLA-DR+ CD4+ T-LYM	7.34 (6.61–8.07)	8.57 (7.33–9.80)	0.120	8.51 (7.67–9.35)	6.30 (5.41–7.19)	**0.005**
CD8+ T-LYM	27.4 (25.8–28.9)	30.7 (28.7–32.7)	**0.010**	29.2 (27.6–30.7)	27.4 (25.5–29.2)	0.286
CD69+ CD8+ T-LYM	6.89 (6.29–7.49)	6.60 (6.03–7.18)	0.908	7.12 (6.54–7.70)	6.12 (5.55–6.69)	0.093
HLA-DR+ CD8+ T-LYM	18.8 (16.8–20.8)	23.3 (19.7–26.8)	0.097	22.8 (20.5–25.2)	15.5 (13.2–17.8)	**0.004**
PD-1+ T-LYM	47.7 (45.5–49.8)	40.8 (38.8–42.9)	**<0.001**	44.0 (42.1–45.9)	47.7 (44.8–50.5)	0.077
CTLA-4+ T-LYM	1.39 (0.84–1.94)	0.36 (0.21–0.50)	**<0.001**	0.85 (0.44–1.25)	1.37 (0.65–2.08)	0.173
B-LYM	10.0 (9.31–10.7)	8.06 (7.32–8.80)	**0.002**	8.88 (8.23–9.54)	10.1 (9.29–11.0)	0.032
Immature B-LYM	5.63 (4.96–6.30)	3.95 (3.03–4.87)	**<0.001**	4.62 (3.97–5.27)	5.85 (4.85–6.84)	**0.035**
NK cells	14.4 (13.3–15.4)	13.9 (12.5–15.2)	0.615	14.8 (13.7–15.8)	13.0 (11.6–14.4)	0.077
Monocytes (MON)	7.72 (7.31–8.12)	8.41 (7.90–8.93)	0.034	8.23 (7.84–8.61)	7.44 (6.90–7.99)	**0.037**
Neutrophils (NEU)	58.5 (57.0–60.1)	56.6 (54.9–58.4)	0.141	58.1 (56.6–59.6)	57.4 (55.4–59.4)	0.711
Eosinophils (EOS)	2.41 (2.07–2.75)	3.01 (2.50–3.53)	**0.034**	2.68 (2.34–3.02)	2.52 (1.98–3.06)	0.286
Basophils (BAS)	0.69 (0.64–0.74)	0.76 (0.70–0.83)	0.069	0.72 (0.67–0.77)	0.72 (0.64–0.79)	0.628
	**(e) Positive Serum IgG**			**(f) Positive Serum IgM**		
**Distribution of Immune Cells**	**No**	**Yes**	***p*** **-Value**	**No**	**Yes**	***p*** **-Value**
**[%]**	**Mean (95% CI)**	**Mean (95% CI)**		**Mean (95% CI)**	**Mean (95% CI)**	
Lymphocytes (LYM)	31.6 (28.6–34.6)	30.4 (29.3–31.5)	0.814	31.2 (29.5–32.9)	30.1 (28.8–31.5)	0.850
CD4+ T-LYM	43.6 (40.5–46.7)	44.4 (43.1–45.7)	0.831	43.0 (41.2–44.8)	45.0 (43.5–46.6)	0.084
CD69+ CD4+ T-LYM	4.54 (2.90–6.18)	6.82 (6.18–7.46)	**0.002**	4.50 (3.69–5.31)	7.70 (6.91–8.48)	**<0.001**
HLA-DR+ CD4+ T-LYM	5.88 (4.15–7.61)	8.23 (7.52–8.94)	**0.002**	6.74 (5.83–7.65)	8.55 (7.65–9.45)	**0.009**
CD8+ T-LYM	28.5 (25.1–32.0)	28.5 (27.2–29.9)	0.983	28.8 (26.9–30.7)	28.4 (26.8–30.1)	0.662
CD69+ CD8+ T-LYM	6.61 (5.51–7.71)	6.86 (6.38–7.35)	0.983	6.26 (5.65–6.88)	7.15 (6.54–7.75)	0.072
HLA-DR+ CD8+ T-LYM	12.9 (8.42–17.5)	22.0 (20.0–24.0)	**<0.001**	16.7 (14.1–19.3)	23.2 (20.7–25.6)	**0.001**
PD-1+ T-LYM	51.7 (47.1–56.4)	44.2 (42.5–46.0)	**0.005**	49.8 (46.9–52.7)	41.8 (40.0–43.6)	**<0.001**
CTLA-4+ T-LYM	1.85 (-0.08–3.78)	0.87 (0.55–1.18)	0.798	1.64 (0.77–2.50)	0.59 (0.31–0.86)	**<0.001**
B-LYM	9.55 (8.10–11.0)	9.26 (8.67–9.84)	0.937	10.1 (9.27–11.0)	8.76 (8.07–9.44)	**0.009**
Immature B-LYM	5.84 (4.11–7.56)	4.83 (4.24–5.43)	0.273	6.57 (5.55–7.60)	3.99 (3.38–4.60)	**<0.001**
NK cells	14.7 (11.7–17.8)	14.1 (13.2–15.0)	0.983	14.3 (12.8–15.8)	14.2 (13.1–15.3)	0.999
Monocytes (MON)	7.61 (6.45–8.77)	8.08 (7.74–8.41)	0.273	7.29 (6.76–7.81)	8.43 (8.02–8.84)	**0.001**
Neutrophils (NEU)	57.7 (54.1–61.2)	57.9 (56.6–59.2)	0.983	58.1 (56.2–60.0)	57.8 (56.2–59.4)	0.662
Eosinophils (EOS)	2.05 (1.34–2.77)	2.74 (2.42–3.06)	0.067	2.34 (1.80–2.88)	2.82 (2.48–3.17)	**0.010**
Basophils (BAS)	0.63 (0.55–0.71)	0.72 (0.68–0.77)	0.451	0.73 (0.66–0.79)	0.71 (0.65–0.76)	0.666
	**(g) DLCO <80%**			**(g) Gender**		
**Distribution of Immune Cells**	**No**	**Yes**	***p*** **-Value**	**Male**	**Female**	***p*** **-Value**
**[%]**	**Mean (95% CI)**	**Mean (95% CI)**		**Mean (95% CI)**	**Mean (95% CI)**	
Lymphocytes (LYM)	31.6 (30.3–32.9)	29.6 (27.9–31.2)	0.284	30.5 (29.0–32.0)	30.8 (29.4–32.2)	0.692
CD4+ T-LYM	44.6 (43.1–46.1)	44.1 (42.2–46.0)	0.952	42.1 (40.5–43.6)	46.8 (45.2–48.4)	**0.002**
CD69+ CD4+ T-LYM	5.46 (4.69–6.23)	7.27 (6.41–8.13)	**<0.001**	6.46 (5.65–7.27)	6.35 (5.47–7.22)	0.783
HLA-DR+ CD4+ T-LYM	6.59 (5.86–7.32)	9.04 (7.97–10.1)	**<0.001**	8.30 (7.33–9.26)	7.22 (6.37–8.07)	0.218
CD8+ T-LYM	27.8 (26.3–29.4)	29.2 (27.2–31.1)	0.697	29.5 (27.6–31.3)	27.6 (26.1–29.1)	0.594
CD69+ CD8+ T-LYM	6.23 (5.79–6.67)	7.40 (6.63–8.18)	0.078	6.36 (5.85–6.87)	7.25 (6.54–7.97)	0.186
HLA-DR+ CD8+ T-LYM	16.3 (14.3–18.2)	24.8 (21.8–27.8)	**<0.001**	21.2 (18.6–23.9)	19.5 (17.0–22.0)	0.692
PD-1+ T-LYM	47.6 (45.2–49.9)	42.9 (40.7–45.0)	**0.018**	43.8 (41.6–45.9)	46.8 (44.5–49.2)	0.186
CTLA-4+ T-LYM	1.32 (0.72–1.93)	0.71 (0.33–1.08)	**0.039**	1.06 (0.52–1.59)	0.98 (0.49–1.46)	0.770
B-LYM	9.90 (9.25–10.5)	8.75 (7.91–9.60)	**0.012**	8.88 (8.16–9.59)	9.7 (8.98–10.5)	0.285
Immature B-LYM	5.93 (5.15–6.70)	4.13 (3.38–4.88)	**<0.001**	4.81 (4.08–5.54)	5.26 (4.43–6.08)	0.692
NK cells	14.2 (13.0–15.3)	14.2 (12.9–15.5)	0.839	15.8 (14.5–17.0)	12.5 (11.4–13.6)	**0.002**
Monocytes (MON)	7.66 (7.25–8.07)	8.28 (7.78–8.79)	0.194	8.51 (8.03–8.99)	7.38 (6.99–7.76)	**0.007**
Neutrophils (NEU)	57.3 (55.9–58.8)	58.5 (56.5–60.4)	0.790	57.5 (55.7–59.2)	58.3 (56.7–59.8)	0.783
Eosinophils (EOS)	2.38 (2.06–2.71)	2.81 (2.32–3.29)	0.346	2.64 (2.28–3.00)	2.61 (2.16–3.06)	0.654
Basophils (BAS)	0.70 (0.64–0.76)	0.74 (0.68–0.81)	0.219	0.71 (0.65–0.77)	0.72 (0.66–0.78)	0.783

LYM: lymphocytes; MON: monocytes; NEU: neutrophils; EOS: eosinophils; BAS: basophils; DLCO: carbon monoxide diffusing capacity. The data are presented as the mean values and the 95% CI (in brackets).

## Data Availability

Data are available from the corresponding author upon reasonable request.
